# The Progress in Exploratory Studies of Peripheral Blood Single Nucleated Cells as Seed Cells in Peripheral Nerve Repair

**DOI:** 10.1155/np/8895041

**Published:** 2025-08-07

**Authors:** Yi Chen, Yubin Wen, Mingyue Zhang, Jing Nie, Guangfeng Sun

**Affiliations:** ^1^Department of Burns and Plastic Surgery, The Second Affiliated Hospital of Zunyi Medical University, Zunyi, Guizhou, China; ^2^Key Laboratory of Basic Pharmacology of Ministry of Education and Joint International Research Laboratory of Ethnomedicine of Ministry of Education, Zunyi Medical University, Zunyi, Guizhou, China

**Keywords:** nerve generation, PBMC, peripheral blood mononuclear cells, peripheral nerve injury

## Abstract

Peripheral nerve injury (PNI) represents a prevalent clinical condition, often resulting from mechanical trauma or tumor resection, which frequently induces persistent sensory deficits, motor impairment, neuropathic pain, or paralysis. Consequently, substantial socioeconomic burdens are imposed on affected individuals. Autologous nerve transplantation is often considered the preferred approach for reconstructing peripheral nerve defects; however, this technique is associated with limitations including donor-site sensory loss, restricted graft length, and nerve mismatches. Recently, peripheral blood mononuclear cells (PBMCs) have emerged as a focal point in nerve regeneration research due to their accessibility, immunomodulatory properties, and neuro-reparative potential. Nevertheless, the precise mechanisms underlying PBMC-mediated nerve repair remain incompletely characterized, and their molecular pathways require further elucidation. This study explores the potential role of PBMCs in promoting peripheral nerve regeneration, with a particular focus on their regulation of retrograde brain-derived neurotrophic factor (BDNF) transport through modulation of Hook1 expression and associated molecular pathways. This research seeks to provide novel insights for PBMC-based therapeutic strategies and establish a theoretical foundation for clinical translation. Implementation challenges and translational prospects for PBMCs in nerve regeneration are also critically evaluated.

## 1. Introduction

Peripheral nerve injury (PNI) is a common clinical trauma. Epidemiological studies show that the annual incidence of PNI in affluent nations ranges from 13 to 23 per 100,000 individuals [1], while 8% of neurotrauma cases are medically induced, contributing to an estimated 25 million peripheral injuries worldwide each year [2]. Currently, peripheral nerve injuries are mainly caused by mechanical trauma, surgery, or disease complications (e.g., infection, tumor resection) [3]. The nature and severity of the injury directly determines the clinical presentation, with typical symptoms including deficits in sensory and motor function, which significantly impair the patient's quality of life [4].

At present, the primary surgical approaches for repairing PNI include direct neurorrhaphy, autologous nerve transplantation, and artificial nerve conduit implantation [5]. End-to-end neurorrhaphy is commonly employed to repair short-segment nerve defects ( <1 cm), and its success rate depends on the precision of end-to-end alignment. However, when the nerve defect exceeds 1 cm, traditional microsurgical technique often fail to achieve the desired repair outcome [6]. Although, autologous nerve grafts are considered the gold standard for treating peripheral nerve defects [7], their clinical effectiveness remains limited due to donor source con straints [8], postoperative secondary injury [9], and immune rejection [10]. While nerve conduits, have demonstrated efficacy in repairing short-segment nerve defects, their effectiveness in bridging long nerve gaps ( >3 cm) remains limited [4]. Cell therapy has made considerable progress in recent years as a crucial method for repairing peripheral nerve injuries. This includes stem cell transplantation, Schwann cell (SC) transplantation, and immune cell therapy, etc. [11]. Among these, SC transplantation has attracted significant attention for its role in PNI repair. Schwann cells (SCs) facilitate nerve regeneration by providing direct nutritional support, clearing myelin debris and upregulating proteins related to axon regeneration [12]. However, the limited availability of SCs and their declining reparative capacity over time may hinder the effectiveness of nerve regeneration [13]. As a result, identifying more durable and effective cell sources and treatment strategies remains a primary focus of current research. Stem cells have gained considerable attention in nerve injury repair owing to their high proliferative capacity and multipotent differentiation potential. Adipose-derived mesenchymal stem cells (ADSCs) [14], bone marrow-derived mesenchymal stem cells (MSCs) [15], and human embryonic stem cells (ESCs) [16] have shown promise in promoting neural regeneration in animal models. However, the clinical translation of stem cell therapy faces numerous challenges, including difficulties in cell harvesting, low survival rates posttransplant, immune rejection, and ethical concerns [17–19]. Therefore, there is a need to identify seed cells that are relatively safe and easily accessible to provide effective therapies for the repair and reconstruction of PNI. Previous studies have demonstrated that peripheral blood mononuclear cells (PBMCs) play a role in the repair of PNI [20]. However, the specific mechanisms by which PBMCs regulate nerve regeneration, especially the underlying molecular signaling pathways, remain poorly understood. Most existing research has focused on the phenotypic effects of PBMCs on nerve function and tissue repair, while the intracellular signaling pathways involved remain underexplored. Therefore, this review aims to elucidate the mechanisms by which PBMCs act as seed cells in peripheral nerve repair, to investigate their multiple roles in nerve repair, and to summarize the current research progress, as well as, future directions and existing challenges. Through an in-depth analysis of the molecular hypothesis regarding PBMCs in peripheral nerve repair, this review seek to provide new theoretical foundations and research directions for the treatment of PNIs and facilitate the translation of this field into clinical practice.

## 2. Classification and Regeneration Mechanism of PNI

### 2.1. Classification

Seddon (1943) first classified peripheral nerve injuries into three types [21]. Neurapraxia is the mildest form and is characterized by a transient conduction block, typically caused by mild traction or compression. In these injuries, the nerve fibers' anatomical integrity is preserved, and Wallerian degeneration does not occur. Neurological function generally recovers spontaneously within days to weeks, resembling recovery after “shock”. Axonotmesis is more severe than neurapraxia. Here, axons are disrupted within the myelin sheath, but the nerve sheath remains intact. Distal nerve fibers undergo Wallerian degeneration, while proximal axons retain regenerative capacity and gradually restore function along the original path. This repair process may take weeks to months. Neurotmesis is the most severe type, involving complete severance of the nerve trunk, necessitating surgical repair. Wallerian degeneration occurs distal to the injury. The regeneration process is complex and challenging, often resulting in incomplete functional recovery, similar to the difficulty of repairing a broken bridge. Sunderland [22] further refined this system in 1968. He classified nerve injury into five degrees based on the alterations observed in the normal nerve structure. These degrees are (i) damage to the myelin sheath only; axons remain intact without wallerian degeneration, allowing neurological function to recover within weeks, (ii) disruption of axonal continuity with intact endoneurial tubes; distal wallerian degeneration occurs, (iii) damage to both endoneurial tubes and axons, with distal wallerian degeneration complicating internal repair, (iv) damage to nerve fascicles with only the epineurium intact, necessitating surgical intervention, and (v) complete nerve transection including axons and connective tissue, often resulting in poor functional recovery.

### 2.2. Neurotization

Neurological recovery depends on multiple factors, including the severity and location of the nerve injury as well as the patient's physiological condition [23]. Following PNI, the distal segment undergoes a multistep repair process involving Wallerian degeneration, activation and proliferation of SCs, axonal regeneration, and reinnervation of target organs [24]. Wallerian degeneration refers to a series of changes occurring in distal nerve fibers following axonal injury [25]. It is characterized by degeneration and fragmentation of distal axons and myelin sheaths [26], which are subsequently cleared by phagocytosis performed by SCs and infiltrating macrophages [27, 28]. SCs are activated after nerve injury and proliferate to form neural membrane tubes. Phenotypic changes occur, and SCs elongate through the injury gap to form a Büngner' s band that supports the passage of the proximal growth cone [29]. SCs secrete several neurotrophic factors, including glial cell line-derived neurotrophic factor (GDNF), brain-derived neurotrophic factor (BDNF), nerve growth factor (NGF), and neurotrophin-3 (NT-3), which guide regenerating axons along appropriate pathways [30]. The proximal end of the injured axon begins to sprout and grow along the Büngner's band formed by SCs. When the regenerating axon reaches the target organ, SCs form a new myelin sheath around the axon, restoring the integrity of the nerve fiber [31]. This process is analogous to rewiring a circuit to ensure that the various organs are reconnected, and it is a critical step in restoring normal nerve conduction function. Target organs undergo a series of changes after nerve injury, such as muscle atrophy and sensory loss [32]. Therefore, timely repair is essential to maximize the regenerative potential of the intrinsic mechanism of nerve regeneration and to prevent permanent end-organ loss.

## 3. The Composition of PBMCs and Their Role In Peripheral Nerve Repair

### 3.1. Cell Composition of PBMCs

PBMCs consist of several classes of immune cells, including lymphocytes (T cells, B cells, and natural killer (NK) cells), monocytes, and dendritic cells [33]. In humans, the frequencies of these cell types vary between individuals. Typically, lymphocytes constitute 70%–90% of the total population, monocytes account for 10%–20%, and dendritic cells are rare, comprising only 1%–2%. Within the lymphocyte population, T cells represent 70%–85%, B cells make up 5%–10%, and NK cells account for 5%–20% (Verhoeckx, Cotter, and López-Expósito, 2015)

### 3.2. The Role and Mechanism of PBMCs in Peripheral Nerve Repair

T cells are involved in the regulation of myelin regeneration, neuropathic pain, and the immune microenvironment by releasing cytokine, including interferon-gamma (IFN-γ) and interleukin-4 (IL-4), which aid in PNI repair [34]. B cells regulate the immune reaction after nerve injury by secreting antibodies and increasing cytokine activity [35]. Dendritic cells are another type of cell in PBMCs. They help maintain immune balance in the injured area by activating the immune response and interacting with T cells, thereby providing a favorable microenvironment for nerve repair and regeneration [36]. It has been shown that when dendritic cells are stimulated by antigens, the expression of NT, such as NT-3 and BDNF is upregulated [37], which play crucial roles in the development and survival of the nervous system. NK cells in PBMCs can kill damaged and necrotic cells following nerve injury, inhibit inflammatory response, and create a suitable environment for nerve regeneration [38]. Their surface activation receptors enable NK cells to identify molecular patterns on damaged cells, activate intracellular signaling pathways, release cytotoxic chemicals, such as perforin and granzyme, and rapidly clear these abnormal cells [39]. Futhermore, NK cells can also produce various cytokines, such as interferon-γ (IFN-γ) and tumor necrosis factor-α (TNF-α), which play a role in regulating the immune response and nerve repair [40]. Compared to lymphocytes, monocytes vary in complexity and size. They circulate in the blood to peripheral tissues, where they can migrate to the site of injury and differentiate into macrophages. Macrophages play a crucial role in clearing necrotic tissue and promoting nerve regeneration. Monocytes in PBMCs can migrate to the injured area and transform into macrophages, which help eliminate necrotic tissue and facilitate nerve regeneration [41]. M2 macrophages produce cytokines, such as IL-10, IL-4, IL-13, transforming growth factor-β (TGF-β) and NGF. They inhibit the pro-inflammatory effect of M1 macrophages and prevent neuronal apoptosis and promote nerve repair [42]. Furthermore, activated macrophages can also secrete several growth factors, such as NGF, BDNF, and vascular endothelial growth factor (VEGF) [43], which are essential for neuronal survival and axon regeneration. NGF induces autophagy in dedifferentiated SCs, accelerate the clearance and phagocytosis of myelin debris, and promote the regeneration of damaged axon and myelin sheaths in the early stages of PNI [44]. Studies have shown that the application of exogenous BDNF after sciatic nerve transection may increase axons diameter and the thickness of the myelin sheath, facilitating the restoration of neurological function [45]. VEGF is also critical for the revascularization of regenerating nerves. It enhances the proliferation and migration of SCs after PNI, promoting nerve injury repair [46]. Lymphocytes in PBMCs play a significant role in the repair of PNI.

PBMCs not only play a direct role in nerve repair but also participate in myelin repair processes by differentiating into other cell types, such as oligodendrocyte precursor cells (OPCs). Studies have shown that it is feasible to reprogram adult PBMCs into OPCs in vitro using nonintegrated plasmid vectors. These cells exhibit proliferative capacity and can continue to differentiate into mature oligodendrocytes. By expressing key myelin-related proteins, such as MBP, they significantly promote myelin regeneration and improve neurological function [47]. Although the role of PBMCs in the repair of PNI has garnered increasing attention, the specific mechanisms underlying their effects remain incompletely understood. Existing research primarily focuses on how PBMCs promote nerve repair through immune regulation, cell differentiation, and pro-inflammatory/anti-inflammatory mechanisms. Their clinical application, long-term effects and potential synergistic effects with other repair strategies still require further exploration.

### 3.3. Comparison of PBMCs and Stem Cells

Compared to traditional stem cell types, PBMCs offer several significant advantages in clinical applications. First, PBMCs can be easily obtained through a simple blood draw, eliminating the need for invasive surgery or complex cell culture procedures [48]. This extraction method causes minimal harm to the patient and raises no ethical concerns [49]. The process typically involves conventional density gradient centrifugation and does not require complex in vitro amplification or culture, making it highly suitable for clinical use. In contrast, MSCs are usually harvested from bone marrow or adipose tissue through invasive procedures, and their culture process is more complex. Neural stem cells (NSCs) are even more challenging to obtain and culture, often requiring specialized techniques to extract them from the brain or spinal cord. Additionally, since PBMCs are derived from the patient's own body, they exhibit excellent immune compatibility, thereby eliminating the risk of immune rejection—a common issue with traditional stem cell sources, particularly in allogeneic transplants. At the same time, PBMCs lack undifferentiated cells, thus avoiding the risk of teratoma formation associated with stem cells, especially ESCs and induced pluripotent stem cells (iPSCs) [50]. Studies have shown that, compared to bone marrow-derived stem cells, PBMCs—especially those mobilized from peripheral blood—are less likely to be contaminated by malignant cells. This characteristic is particularly crucial for patients with hematological malignancies, as it reduces the risk of reintroducing cancer cells during treatment [51]. Moreover, PBMCs play a key role in immune regulation and can facilitate nerve repair by suppressing excessive inflammation and promoting the secretion of neurotrophic factors, such as BDNF and NGF. Consequently, PBMCs have broad potential for use in peripheral nerve repair. Given these advantages, PBMCs are especially well-suited for early intervention in acute nerve injuries and for treating specific populations, such as cancer patients, requiring nerve repair.

## 4. The Clinical Application and Research Progress of PBMCs

In our earlier research, we conducted preliminary exploratory experiments to evaluate the therapeutic potential of PBMCs in peripheral nerve repair. PBMCs were isolated from Sprague-Dawley (SD) rats using polysucrose-glucosamine diatrizoate density gradient centrifugation. A complete sciatic nerve transection model was established in SD rats, followed by end-to-end epineural anastomosis. At the site of anastomotic, a PBMC-containing cell suspension was applied locally [52].

Preliminary findings from the PBMC-treated group revealed the following trends: (1) improved toe extension claw reflex, (2) increased sciatic nerve function index (SFI), (3) greater wet weight of the gastrocnemius muscle on the injured side, and (4) enhanced regeneration of myelinated nerve fibers at the distal stump. These results suggest that PBMCs may contribu to functional recovery after sciatic nerve injury, although larger-scale studies are necessary to confirm these effects.

This therapeutic approach was also applied clinically. Between April 2011 and September 2015, autologous PBMCs were administered to 12 patients with mid-upper arm radical nerve injuries. After microscopic end-to-end epineural suturing, the anastomotic site was covered with an absorbable gelatin sponge soaked in 5 mL of autologous PBMC suspension, while the remaining 10 mL was injected into surrounding damaged muscle tissue. Following a 15–36 month follow-up period, most patients exhibited improved wrist and finger motor functions [53], suggesting that PBMCs may also have therapeutic value in human peripheral nerve repair.

To explore the underlying mechanisms, we assessed spinal cord neuronal apoptosis and BDNF expression in the spinal cord. After sciatic nerve transection and repair, no significant difference in the number of apoptotic cells was observed between the transection-only and anastomosis groups; however, both showed markedly increased apoptosis compared to the sham group [49]. In contrast, the PBMC-treated group exhibited a notable reduction in apoptotic cells within the anterior horn of the spinal cord and showed relatively preserved neuronal morphology, suggesting a potential neuroprotective effect of PBMCs on motor neurons.

**Figure 1:**
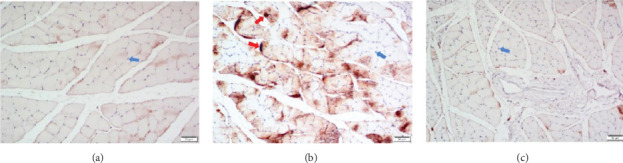
Expression of BDNF protein in the gastrocnemius muscle of rats. (a) Sham group, (b) control group, and (c) PBMCs group.

BDNF, a key NT expressed in spinal anterior horn cells, is primarily transported retrogradely from peripheral target organs. In our study, BDNF expression in the spinal cord was reduced in the anastomosis group but increased in the PBMC-treated group. Furthermore, as shown in Figure 1, BDNF levels in the gastrocnemius muscle were elevated 1 month postoperatively in the model group, but decreased in the PBMC group. This inverse relationship may indicate that PBMCs promote retrograde BDNF transport from peripheral tissues to the spinal cord, thereby supporting neuronal survival.The elevated BDNF levels observed in the gastrocnemius muscle of the model group may be attribute to impaired retrograde transport following sciatic nerve injury. When axonal transport is disrupted, BDNF produced in peripheral tissues may fail to reach anterior horn neurons, resulting in its accumulation at the production site. This accumulation may represent a compensatory response of denervated muscle, where local upregulation of neurotrophic factors occurs as an adaptive mechanism to nerve injury [54].

The blue arrow points to the cell nuclei, which appear purple blue (Hematoxylin staining). The red arrow points to the BDNF expression region, which appears brown (DAB staining).

This preliminary observation may suggests a possible involvement of PBMCs in modulating the BDNF transport process. While a previous study [55] reported a reduction in BDNF expression in the spinal cord after nerve injury, supporting the potential role of the BDNF signaling pathway in PBMC-mediated neuroprotection, our current findings are based on limited sample size and qualitative immunohistochemical analysis. Therefore, further molecular-level and quantitative investigations are required to validate this hypothesis. The scientific hypothesis of PBMCs repairing PNI.

## 5. The Scientific Hypothesis of PBMCs Repairing PNI

BDNF is a key regulator of neuronal survival, axonal growth, synaptic plasticity, and neural repair [56]. Upon binding to its receptor TrkB, BDNF induces TrkB dimerization, and tyrosine autophosphorylation, initiating a cascade of downstream signaling pathways, including the MAPK/ERK pathway, PI3K/Akt pathway, and PLC-γ signaling [57].

### 5.1. The Role of Rab Proteins in TrkB Endocytosis and Transport

Following BDNF binding, the TrkB receptor is not retained at the cell surface but undergoes endocytosis [58, 59] and is transported retrogradely along the axon to the soma, where it activates the expression of repair-related genes. The intracellular trafficking is regulated by the monomeric GTPase Rab family [60]. Rab5, a small GTPase, is central to the early endocytosis of TrkB and serves as a marker for early endosomes [61]. TrkB endocytosis is specifically mediated by Rab5 [62]. After activation by BDNF in both dendrites and the soma, TrkB localizes to Rab5- and Rab11-positive endosomes. These Rab5-positive endosomes not only act as carriers for TrkB transport but also preserve its downstream signaling activity, particularly via the PI3K/Akt pathway [63]. Sustained BDNF signaling requires the coordinated activity of Rab5 and Rab11. Rab11, in particular, is essential for dendritic branching and local signaling in dendrites and synapses [64].

At injured nerve terminals, Rab5 activation facilitates TrkB receptor endocytosis and determines the receptor's fate. Conversion of Rab5 to Rab7 typically directs TrkB toward lysosomal degradation, whereas Rab5-mediated trafficking to Rab11-positive endosomes allows receptor recycling back to the plasma membrane, thereby maintaining signaling continuity [65].

### 5.2. Microtubule-Dependent Retrograde Transport of BDNF-TrkB Endosomes

BDNF-TrkB signaling endosomes are transported retrogradely from the synaptic terminal to the soma via axonal microtubles. This process depends not only on microtubules as structural tracks but also on motor proteins, such as kinesin and cytoplasmic dynein [66]. Dynein is the principal motor protein responsible for retrograde transport, enhancing efficiency and stability through the formation of a dynactin–dynein complex [67–69]. In mammals, the interaction between dynein and dynactin is modulated by multiple cofactors, including Bicaudal D2 (BICD2) [70], Spindly [71], Hook [72], and Rab11/FIP3 [73].

### 5.3. The Role and Regulation of Hook1

**Figure 2:**
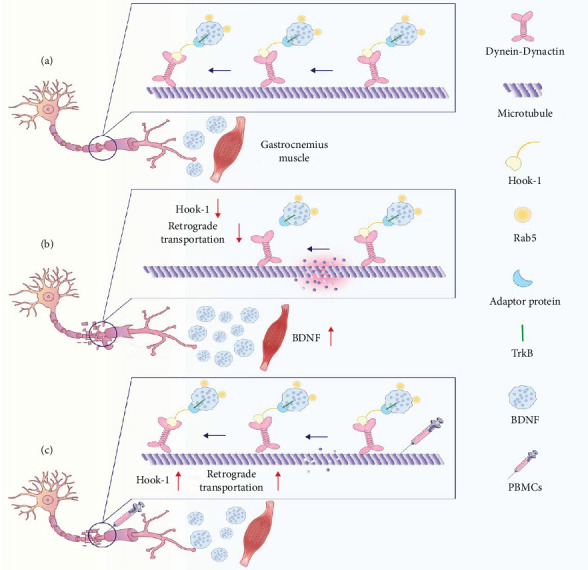
Scientific hypothesis of PBMCs repairing peripheral nerve injury. (a) Hook1-mediated BDNF/TrkB retrograde axonal transport under normal conditions. (b) The decreased expression of Hook1 after nerve injury leads to impaired BDNF/TrkB retrograde transport. (c) Local treatment of PBMCs restored Hook1 expression, promoted BDNF/TrkB retrograde transport and nerve repair.

Hook1 is a microtubule-associated protein that interacts with the dynein-dynactin complex to enhance its stability and motility along microtubules [74]. Beyond its role in intracellular transport, Hook1 is also involved in the endocytosis and retrograde trafficking of BDNF/TrkB receptor complexes, thereby modulating both the duration and intensity of downstream neurotrophic signaling [75]. Studies have shown that Hook1 co-migrates with TrkB and Rab5-positive endosomal subpopulations carrying BDNF, which exhibit increased retrograde mobility compared to the broader Rab5-positive population. Knockout of Hook1 results in widespread disruption of BDNF signaling endosomes transport, without affecting the transport of other organelles [76]. Additionally, active Rab5 can form a complex with Hook1-interacting proteins, which associate with dynein and actin to regulate the retrograde axonal transport of neuronal proteins [77]. Following PNI, decreased expression of Hook1 and Rab5 impairs the retrograde transport of TrkB receptors, disrupting the transmission of repair signals and contributing to neuronal dysfunction. In our previous study, proteomic analysis of the proximal nerve segment in a rat sciatic nerve transection model revealed significantly lower Hook1 protein levels 1 month after sciatic nerve anastomosis compared to the control group. However, Hook1 levels were notably elevated in the PBMC-treated group [78]. We speculate that PBMCs may enhance nerve repair by upregulating Hook1 expression, thereby improving the retrograde transport efficiency of the BDNF/TrkB complex. In peripheral nerve repair, BDNF is retrogradely transported from the damaged nerve terminals to the neuronal soma, where it activates key signaling pathways involved in regeneration. Based on preliminary data, we speculate that PBMCs may promote this transport process by upregulating Hook1 expression (Figure 2). However, as our current analysis is based on a small number of samples and lacks quantitative validation, these findings should be interpreted as early observations that require further confirmation in future studies.

## 6. Challenges and Prospects

Although PBMCs have shown promising regenerative potential in animal models, several challenges must be before clinical translation is feasible. First, protocols for PBMC isolation and expansion require further refinement to improve cell purity and viability. Additionally, the efficiency of in vitro differentiation into neurons or SC-like phenotypes remains suboptimal. Enhancing lineage-specific induction and optimizing differentiation conditions are essential directions for future research. Second, the underlying mechanisms by which PBMCs exert their therapeutic effects remain incompletely understood and warrant further investigation. Although multiple hypotheses have been proposed to explain how PBMCs facilitate peripheral nerve repair, these theories require more rigorous experimental validation. In-depth mechanistic studies are crucial for elucidating the role of PBMCs in nerve regeneration and for guiding their clinical application with greater scientific precision. Currently, clinical research on PBMCs for PNI is in its early stages, and large-scale, long-term data are lacking. Therefore, expanded clinical trials with extended follow-up are needed to comprehensively assess the therapeutic value of PBMCs in nerve repair. In the future, continued technological and scientific advancements may position PBMCs as a core component in the treatment of peripheral nerve injuries. At the same time, interdisciplinary collaboration and translational research will be vital for accelerating the clinical application of PBMC-based therapies. Leveraging such collaborations, PBMCs could be combined with other therapeutic strategies to generate synergistic effects and further enhance outcomes. Translation research will play a critical role in converting basic discoveries results into effective clinical interventions, ultimately expanding the therapeutic arsenal available to patients. It is anticipated that PBMCs will play an increasingly prominent role in the field of peripheral nerve repair, offering new hope to patients affected by nerve injuries.

## 7. Conclusion

PNI is common clinical condition, with various treatment strategies tailored to different injury types. PBMCs have emerged as promising seed cells for nerve repair. Preclinical evidence suggests that PBMCs may promote neuromuscular recovery in rats with sciatic nerve transection and exert protective effects on spinal motor neurons in the anterior horn. Although preliminary observations suggest that PBMCs may influence retrograde axoplasmic transport of BDNF, whether PBMCs facilitate the retrograde axonal transport of BDNF signaling to the neuronal soma in sciatic nerve axons by enhancing Hook1 expression, thereby contributing to neuromuscular recovery, remains to be fully elucidated and requires further investigation.

With systematic exploration of the underlying biological pathways and continued refinement of clinical strategies, PBMCs hold potental as a novel therapeutic modality for peripheral nerve injuries. Future progress will depend on close interdisciplinary collaboration and the advancement of clinical translational research to clarify and expand their clinical utility.

## Data Availability

Data sharing is not applicable to this article as no datasets were generated or analyzed during the current study.
